# Dopaminergic signaling within the primary cilia in the renovascular system

**DOI:** 10.3389/fphys.2015.00103

**Published:** 2015-04-16

**Authors:** Kimberly F. Atkinson, Sarmed H. Kathem, Xingjian Jin, Brian S. Muntean, Wissam A. Abou-Alaiwi, Andromeda M. Nauli, Surya M. Nauli

**Affiliations:** ^1^Department of Biomedical and Pharmaceutical Sciences, Chapman UniversityIrvine, CA, USA; ^2^Department of Pharmacology and Experimental Therapeutics, University of ToledoToledo, OH, USA; ^3^Department of Pharmaceutical and Biomedical Sciences, California Northstate UniversityElk Grove, CA, USA

**Keywords:** ciliopathy, ciliotherapy, cilium, blood pressure, polycystic kidney disease

## Abstract

Activation of dopamine receptor type-5 (DR5) has been known to reduce systemic blood pressure, most likely by increasing renal vasodilation and enhancing natriuresis in the kidney. However, the mechanism of DR5 in natriuresis and vasodilation was not clearly known. We have previously shown that DR5 is localized to primary cilia of proximal renal epithelial and vascular endothelial cells. We here show that selective activation of DR5 specifically induces calcium influx only in the primary cilia, whereas non-selective activation of dopamine receptor induces calcium fluxes in both cilioplasm and cytoplasm. Cilia-independent signaling induced by thrombin only shows calcium signaling within cytoplasm. Furthermore, calcium activation in the cilioplasm by DR5 increases length and mechanosensory function of primary cilia, leading to a greater response to fluid-shear stress. We therefore propose a new mechanism by which DR5 induces vasodilation via chemical and mechanical properties that are specific to primary cilia.

## Sensory functions of primary cilia

The primary cilium is a non-motile sensory organelle that extends from the apical cell surface, functioning as a sensory antenna in most mammalian and eukaryotic cells. Fluid mechanosensing by cilia allows cells to sense urine flow in the kidney, blood flow in the vasculature, hepatic bile in the liver, digestive fluid in the pancreas, lacunocanalicular fluid in bone or cartilage, nodal flow in Hensen's node, and many others (Marshall and Nonaka, 2006). Recently, our laboratory has also found a specific-chemical sensing role of **primary cilia** in both vascular endothelia and renal epithelia (Kathem et al., 2014; Upadhyay et al., 2014).

KEY CONCEPT 1 **Primary cilia**

A cilium is a finger-like projection from the cell surface consisting of a microtubule cytoskeleton with a modified cell membrane. The development and function of cilia is likely determined by 200 genes conserved in all ciliated cells and absent in non-ciliated cells (Tomer et al., 2004). The primary cilium is a solitary non-motile organelle lacking a central pair of microtubules that exist in motile cilia, and is highly conserved in eukaryotic cells. Aside from genetic abnormalities, manipulating micro-environment mechanically and chemically are probably the most used cues to study cilia functions. Along with mechanosensory (ex. fluid flow) and chemosensory (ex. fenoldopam) functions, primary cilia have been proposed to have other physiological roles (Jin et al., 2014).

The clinical importance of primary cilia has only begun to be understood. Dysfunctional cilia are recently thought to be the cause of a group of diseases termed “ciliopathies,” which include polycystic kidney disease (PKD), Bardet-Biedl Syndrome, primary ciliary dyskinesia, nephronophthisis, and many others (Marshall and Nonaka, 2006; Winyard and Jenkins, 2011). Cellular loss of sensory input from the antennae-like primary cilium renders the cell unresponsive to environmental cues. The mechanosensation of primary cilia occurs primarily in response to fluid-shear stress, while chemosensation refers to the cells' ability to sense chemical cues such as ligands in the blood or neurotransmitters in the synapse (Muntean et al., 2014).

Determining the sensory and molecular properties of primary cilia is therefore key to understanding dysfunction or abnormal development of cilia in ciliopathies. For example, in renal cyst development, improper calcium signaling results from abnormal sensory function of cilia to fluid flow (Nauli et al., 2006). Along with intact cilia, the expression and function of polycystins is known to be important in the development of renal cysts (Pazour et al., 2002; Yoder et al., 2002; Nauli et al., 2003). Determining the functions of the receptors and proteins expressed on primary cilia thus becomes very important in the development of potential drug targets in the new pharmaceutical field of “**ciliotherapy**.”

KEY CONCEPT 2 **Ciliopathy**

Ciliopathy refers to a group of diseases caused by mutated proteins within the primary cilium or centrosome, including polycystic kidney disease (PKD), Joubert Syndrome, nephronophthisis, retinitis pigmentosa, among others (Marshall and Nonaka, 2006). Clinical features of ciliopathies include cystic kidney, hypertension, aneurysm, retinal degeneration, polydactyly, cognitive dysfunction, mental retardation, and obesity. The association between cilia and ciliopathy has opened the possibility for cilia targeting therapy (ciliotherapy) (Kathem et al., 2014).

## Ciliary dopamine receptors

**Figure 1:**
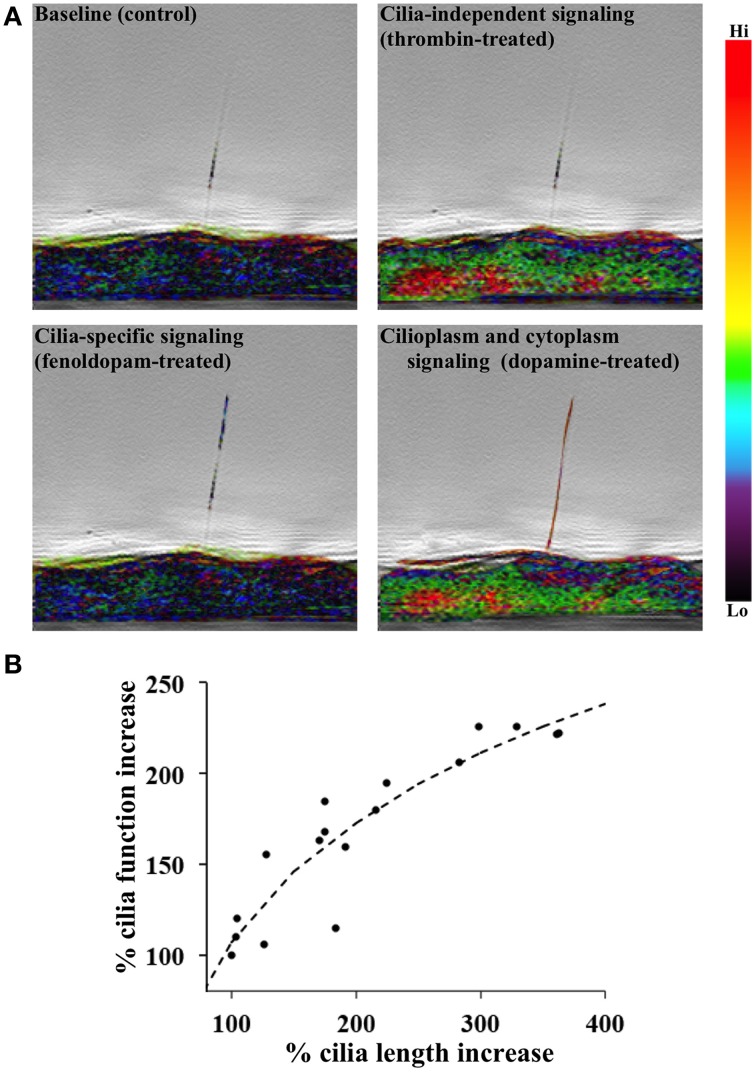
**DR5 activation is associated with primary cilia length-function relationship. (A)** A single endothelium tagged with calcium indicator Gcamp3 was imaged from the side. 10 nM fenoldopam or dopamine was used to selectively or non-selectively activate dopamine receptor type-5 (DR5), respectively. Calcium signaling was specific to cilium in fenoldopam-treated cells but dispersed throughout the dopamine-treated cell. Thrombin (100 nM) represented cilia-independent calcium signaling. Calcium level was pseudo-colored and superimposed with phase images. **(B)** A positive correlation is shown between cilia length and function. Cells were chemically induced with dopamine agonists to provide changes in primary cilia length. Cells were then mechanically challenged with fluid-shear stress to analyze the mechanosensory function of primary cilia using Ca^2+^ signal as a readout. *N* = 28 cell populations for each data point.

Genetic screens of abnormal cilia length have identified a family of class A **dopamine** binding G-protein coupled receptors that are involved in regulation of length of cilia and flagella (Avasthi et al., 2012). Consistent with what we previously demonstrated, treatment of dopaminergic agonists (dopamine or fenoldopam) modulates cilia function by extending cilia length and therefore cilia sensitivity in renal epithelial and vascular endothelial cells (Abdul-Majeed and Nauli, 2011; Kathem et al., 2014). Dopamine receptors are expressed in renal tubules as well as in renal blood vessels, however the dopamine receptor type-5 (DR5) is localized specifically to the primary cilia in these cell types, whereas other dopamine receptors are not expressed or are expressed ubiquitously in the cell membrane (Abdul-Majeed and Nauli, 2011; Kathem et al., 2014). DR5 is therefore likely responsible for binding dopaminergic agonists to transduce calcium signaling in the cilia (Figure 1A). Taken together, these findings suggest that neurological diseases, hypertension and other ciliopathies such as PKD.

KEY CONCEPT 3 **Dopamine**

Dopamine is a circulating hormone and neurotransmitter implicated in hypertension in human and animal models. The roles and functions of dopamine vary in different tissues of the body. The renovascular dopaminergic system is involved in renal blood flow and blood pressure regulation, but its specific regulation is unknown (Upadhyay et al., 2014). dopaminergic agents may be used as potential ciliotherapy for

One of many regulators of cilia length includes the orphan GPR22, a rhodopsin-like GPCR that couples to Gαi/αo to inhibit adenylyl cyclase. GPR22 regulates cilia length and structure as well as left-right symmetry in zebrafish (Verleyen et al., 2014). Another regulator of cilia length is OCRL1 (oculocerebrorenal syndrome of Lowe), a lipid phosphatase that has been shown to modulate cilia length in renal epithelial cells. OCRL1 knockdown leads to elongation of cilia and blunted intracellular Ca^2+^ release in response to ATP (Rbaibi et al., 2012). There are likely multiple regulatory proteins and/or pathways that control cilium length. It has been suggested that cAMP clearance, MAPK signaling, and phosphorylation of ciliary modulator proteins are also involved in the regulation of cilia length by mechanical and chemical stimuli (Abdul-Majeed et al., 2012).

## Cilia length-function relationship

Another topic of interest for research into the treatment of cilia-associated diseases is regulation of cilia length, which is tissue-specific and dependent on extracellular chemical and mechanical stimuli. Renal injury (Verghese et al., 2008), lithium treatment (Wang et al., 2013), among other stimuli, induce elongation of primary cilia (Abdul-Majeed et al., 2012). Fluid-shear stress also alters the mechanosensory properties of cilia (Nauli et al., 2008). When the cilia length-function relationship was analyzed, we show that there was a correlation between cilia length and function (Figure 1B). Cilia length was measured using immunofluorescence staining of acetylated α-tubulin and direct scanning electron microscopy. Ciliary function was then analyzed by cytosolic [Ca^2+^] measurement (Upadhyay et al., 2014). We showed that cilia elongation by dopaminergic activation results in increased cilium function in terms of calcium signaling and NO biosynthesis (Kathem et al., 2014; Upadhyay et al., 2014). Overall, an increase of cilia function would lead to a greater propensity for endothelium-dependent vasodilation in response to fluid-shear stress (Nauli et al., 2008; Aboualaiwi et al., 2009; Lorthioir et al., 2015).

**Figure 2:**
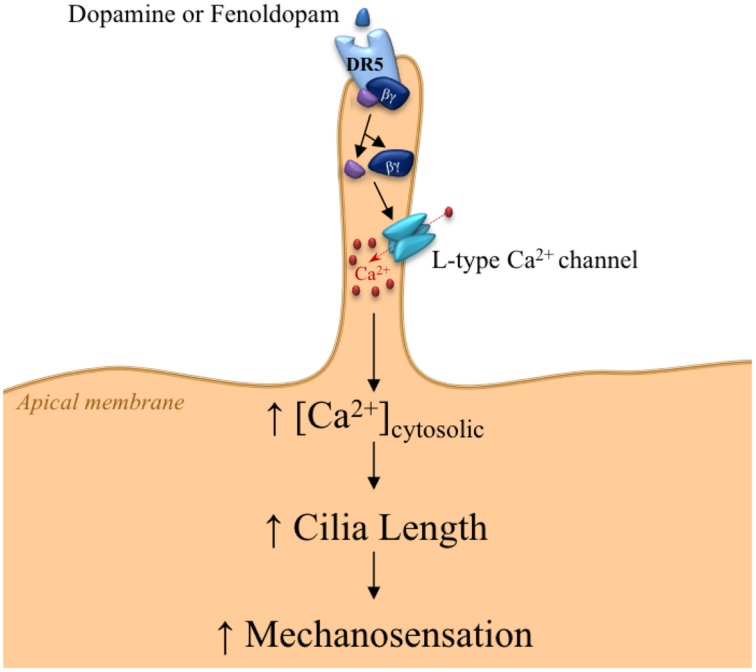
**Proposed mechanism for DR5 signaling within the primary cilium**. The signaling within primary cilia is triggered by the chemosensory function of primary cilia through DR5 activation, resulting in dissociation of Gα and Gβ γ subunits. The Gβ γ subunit activates L-type calcium channels, thereby increasing cilioplasm calcium concentration. Calcium-induced cilia elongation provides greater sensitivity to fluid-shear stress.

In addition, we have provided evidence of dopaminergic signaling involved in sensation by primary cilia through CaV1.2, known as L-type calcium channel (Jin et al., 2014). **DR5** has dual chemo- and mechano-sensory functions within the primary cilia. DR5 activation induces calcium increase in the cilioplasm. Subsequently, DR5-induced calcium channel activation increases length and mechanosensory function of primary cilia, leading to increased calcium signaling and eventual vasodilation in response to fluid-shear stress (Figure 2). This idea emphasizes the importance of ciliary intervention in patients with ciliopathy. Consistent with this idea, we and others have recently shown that ciliary dopaminergic activation increases cilia length and function, further improves outcomes in mouse model *in vivo*, and remedies the vascular markers in ciliopathy (PKD) patients (Kathem et al., 2014; Lorthioir et al., 2015).

KEY CONCEPT 4 **Dopamine receptor type-5 (DR5)**

The expressions of DR5 in renal epithelial and vascular endothelial primary cilia lead us to hypothesize that the mechanism of DR5 involves cilia-specific signaling (Abdul-Majeed and Nauli, 2011). We propose a new mechanism by which DR5 induces vasodilation via chemical and mechanical properties of primary cilia.

## Conclusions

Our current studies suggest that the dopamine receptor DR5 may be a good therapeutic target to manipulate cilia length as well as sensitivity to chemical and mechanical stimuli in ciliopathy. Thus, understanding cilia-specific proteins and signaling pathways is crucial to pharmacologically intervene in the pathogenesis of ciliary diseases. The localization of various proteins within the bulb of a cilium has just been recently understood (Mohieldin et al., 2015). Without doubt, a likely avenue of future research lies in identification and characterization of receptor functions localized to primary cilia of various cell types.

### Conflict of interest statement

The authors declare that the research was conducted in the absence of any commercial or financial relationships that could be construed as a potential conflict of interest.
